# Supplementary material for: Disease-related mutations in PI3Kγ disrupt regulatory C-terminal dynamics and reveal a path to selective inhibitors
Source: eLife. 2021 Mar 4;10:e64691. doi: 10.7554/eLife.64691 (PMC7955810; doi:10.7554/eLife.64691)
Supplement: Supplementary file 1. [file elife-64691-supp1.docx]

| **Data set – Figure 2** | **p110𝛾 / p101** | **R1021C p110𝛾 p101** | **R1021P p110𝛾 p101** |
| --- | --- | --- | --- |
| HDX reaction details | %D_2_O=62.0%  pH_(read)_=7.5  Temp=18ºC | %D_2_O=62.0%  pH_(read)_=7.5  Temp=18ºC | %D_2_O=62.0%  pH_(read)_=7.5  Temp=18ºC |
| HDX time course (seconds) | 3, 30, 300, 3000 | 3, 30, 300, 3000 | 3, 30, 300, 3000 |
| HDX controls | N/A | N/A | N/A |
| Back-exchange | No correction | No correction | No correction |
| Number of peptides | 153 | 153 | 152 |
| Sequence coverage | 92.7% | 92.7% | 91.7% |
| Average peptide  /redundancy | Length=14.8  Redundancy= 2.0 | Length=14.8  Redundancy= 2.0 | Length=14.8  Redundancy= 2.0 |
| Replicates | 3 | 3 | 3 |
| Repeatability | Average StDev=0.6% | Average StDev=0.6% | Average StDev=0.6% |
| Significant differences in HDX | >5% and >0.4 Da and unpaired t-test ≤0.01 | >5% and >0.4 Da and unpaired t-test ≤0.01 | >5% and >0.4 Da and unpaired t-test ≤0.01 |
