# Supplementary material for: Disease-related mutations in PI3Kγ disrupt regulatory C-terminal dynamics and reveal a path to selective inhibitors
Source: eLife. 2021 Mar 4;10:e64691. doi: 10.7554/eLife.64691 (PMC7955810; doi:10.7554/eLife.64691)
Supplement: Supplementary file 2. [file elife-64691-supp2.docx]

| **Data set – Figure 4** | **Apo p110𝛾** | **+ IPI-549** | **+ AZg1/AZ** | | **+ AS-605240** | |
| --- | --- | --- | --- | --- | --- | --- |
| HDX reaction details | %D_2_O=75.5%  pH_(read)_=7.5  Temp=18ºC | %D_2_O=75.5%  pH_(read)_=7.5  Temp=18ºC | %D_2_O=75.5%  pH_(read)_=7.5  Temp=18ºC | | %D_2_O=75.5%  pH_(read)_=7.5  Temp=18ºC | |
| HDX time course (seconds) | 3, 30, 300, 3000 | 3, 30, 300, 3000 | 3, 30, 300, 3000 | | 3, 30, 300, 3000 | |
| HDX controls | N/A | N/A | N/A | | N/A | |
| Back-exchange | Corrected based on %D_2_O | Corrected based on %D_2_O | Corrected based on %D_2_O | | Corrected based on %D_2_O | |
| Number of peptides | 180 | 180 | 180 | | 180 | |
| Sequence coverage | 88.6% | 88.6% | 88.6% | | 88.6% | |
| Average peptide  /redundancy | Length= 13.4  Redundancy= 2.2 | Length= 13.4  Redundancy= 2.2 | Length= 13.4  Redundancy= 2.2 | | Length= 13.4  Redundancy= 2.2 | |
| Replicates | 3 | 3 | 3 | | 3 | |
| Repeatability | Average StDev=0.9% | Average StDev=0.9% | Average StDev=0.9% | | Average StDev=0.9% | |
| Significant differences in HDX | >5% and >0.4 Da and unpaired t-test ≤0.01 | >5% and >0.4 Da and unpaired t-test ≤0.01 | >5% and >0.4 Da and unpaired t-test ≤0.01 | | >5% and >0.4 Da and unpaired t-test ≤0.01 | |
| **Data set – Figure 4** | **+ Gedatolisib** | **+ Omipalisib** | **+ RD-HBC 520** | **+ PIK-90** | |  |
| HDX reaction details | %D_2_O=75.5%  pH_(read)_=7.5  Temp=18ºC | %D_2_O=75.5%  pH_(read)_=7.5  Temp=18ºC | %D_2_O=75.5%  pH_(read)_=7.5  Temp=18ºC | %D_2_O=75.5%  pH_(read)_=7.5  Temp=18ºC | |  |
| HDX time course (seconds) | 3, 30, 300, 3000 | 3, 30, 300, 3000 | 3, 30, 300, 3000 | 3, 30, 300, 3000 | |  |
| HDX controls | N/A | N/A | N/A | N/A | |  |
| Back-exchange | No correction | No correction | No correction | No correction | |  |
| Number of peptides | 180 | 180 | 180 | 180 | |  |
| Sequence coverage | 88.6% | 88.6% | 88.6% | 88.6% | |  |
| Average peptide  /redundancy | Length= 13.4  Redundancy= 2.2 | Length= 13.4  Redundancy= 2.2 | Length= 13.4  Redundancy= 2.2 | Length= 13.4  Redundancy= 2.2 | |  |
| Replicates | 3 | 3 | 3 | 3 | |  |
| Repeatability | Average StDev=0.9% | Average StDev=0.9% | Average StDev=0.9% | Average StDev=0.9% | |  |
| Significant differences in HDX | >5% and >0.4 Da and unpaired t-test ≤0.01 | >5% and >0.4 Da and unpaired t-test ≤0.01 | >5% and >0.4 Da and unpaired t-test ≤0.01 | >5% and >0.4 Da and unpaired t-test ≤0.01 | |  |
