# Supplementary material for: Disease-related mutations in PI3Kγ disrupt regulatory C-terminal dynamics and reveal a path to selective inhibitors
Source: eLife. 2021 Mar 4;10:e64691. doi: 10.7554/eLife.64691 (PMC7955810; doi:10.7554/eLife.64691)
Supplement: Supplementary file 3. [file elife-64691-supp3.docx]

| **Data set – Figure 2- figure supplement 1** | **Apo p110𝛾** | **R1021C** |
| --- | --- | --- |
| HDX reaction details | %D_2_O=87.9%  pH_(read)_=7.5  Temp=18ºC | %D_2_O=87.9%  pH_(read)_=7.5  Temp=18ºC |
| HDX time course (seconds) | 3, 30, 300, 3000 | 3, 30, 300, 3000 |
| HDX controls | N/A | N/A |
| Back-exchange | No correction | No correction |
| Number of peptides | 204 | 202 |
| Sequence coverage | 92.7% | 92.5% |
| Average peptide  /redundancy | Length=14.0  Redundancy= 2.4 | Length=14.0  Redundancy= 2.4 |
| Replicates | 3 (2 3000s, 2 300s) | 3 (2 300s) |
| Repeatability | Average StDev=0.5% | Average StDev=0.5% |
| Significant differences in HDX | >5% and >0.4 Da and unpaired t-test ≤0.01 | >5% and >0.4 Da and unpaired t-test ≤0.01 |
