# Supplementary material for: Disease-related mutations in PI3Kγ disrupt regulatory C-terminal dynamics and reveal a path to selective inhibitors
Source: eLife. 2021 Mar 4;10:e64691. doi: 10.7554/eLife.64691 (PMC7955810; doi:10.7554/eLife.64691)
Supplement: Supplementary file 4. [file elife-64691-supp4.docx]

| **Data set – Figure 4- figure supplement 1** | **Apo p110𝛾/p101** | **+ IPI-549** | **+ Gedatolisib** |
| --- | --- | --- | --- |
| HDX reaction details | %D_2_O=75.5%  pH_(read)_=7.5  Temp=18ºC | %D_2_O=75.5%  pH_(read)_=7.5  Temp=18ºC | %D_2_O=75.5%  pH_(read)_=7.5  Temp=18ºC |
| HDX time course (seconds) | 3, 30, 300, 3000 | 3, 30, 300, 3000 | 3, 30, 300, 3000 |
| HDX controls | N/A | N/A | N/A |
| Back-exchange | No correction | No correction | No correction |
| Number of peptides | 228 | 228 | 228 |
| Sequence coverage | 96.3% | 96.3% | 96.3% |
| Average peptide  /redundancy | Length= 14.1  Redundancy= 2.9 | Length= 14.1  Redundancy= 2.9 | Length= 13.4  Redundancy= 2.2 |
| Replicates | 3 | 3 | 3 |
| Repeatability | Average StDev=0.9% | Average StDev=0.9% | Average StDev=0.9% |
| Significant differences in HDX | >5% and >0.4 Da and unpaired t-test ≤0.01 | >5% and >0.4 Da and unpaired t-test ≤0.01 | >5% and >0.4 Da and unpaired t-test ≤0.01 |
