# Supplementary material for: Disease-related mutations in PI3Kγ disrupt regulatory C-terminal dynamics and reveal a path to selective inhibitors
Source: eLife. 2021 Mar 4;10:e64691. doi: 10.7554/eLife.64691 (PMC7955810; doi:10.7554/eLife.64691)
Supplement: Supplementary file 5. — IC50s for class IA and IB are listed from the reference attached. N.D. is not determined. [file elife-64691-supp5.docx]

|  | Compound | Structure | Reference (PMIDs) | PDB | IC_50_ PI3Kα (nM) | IC_50_ PI3Kβ  (nM) | IC_50_ PI3Kδ  (nM) | IC_50_ PI3Kγ  (nM) |
| --- | --- | --- | --- | --- | --- | --- | --- | --- |
| 1 | IPI-549 |  | 27660692,  32865410 | This study and 6XRL | 3,200 | 3,500 | >8,400 | 16 |
| 2 | PIK-90 |  | 19318683 | 2CHX | 11 | 350 | 58 | 18 |
| 3 | AS-604850 |  | 16127437 | 2A4Z | 4,500 | >20,000 | >20,000 | 250 |
| 4 | Gedatolisib  PF-05212384  PKI587 |  | 20166697 | This study | 0.4 | - | - | 5.4 |
| 5 | Omipalisib (GSK2126458, GSK458) |  | 24900173 | 3L08 | 0.0019 (*K*_i_) | 0.13 (*K*_i_) | 0.024 (*K*_i_) | 0.06 (*K*_i_) |
| 6 | NVS-PI3-4 |  | 23029326 | This study | 1,800 | 250 | 750 | 90 |
| 7 | AZ2 |  | 30718815 | N.D. | 3,981 | 31,622 | 200 | 0.3 |
