# Supplementary material for: Disease-related mutations in PI3Kγ disrupt regulatory C-terminal dynamics and reveal a path to selective inhibitors
Source: eLife. 2021 Mar 4;10:e64691. doi: 10.7554/eLife.64691 (PMC7955810; doi:10.7554/eLife.64691)
Supplement: Supplementary file 6. [file elife-64691-supp6.docx]

**Table S6** **X-ray** **Data collection and refinement statistics**

|  | PI3Kγ  IPI549  PDB:7JWZ | PI3Kγ  Gedatolisib  PDB:7JWE | PI3Kγ  NVS-PI3-4  PDB:7JX0 |
| --- | --- | --- | --- |
| **Data collection** |  |  |  |
| Wavelength | 0.97949 | 0.97949 | 0.97949 |
| Space group | C121 | C121 | C121 |
| Cell dimensions |  |  |  |
| *a*, *b*, *c* (Å) | 144.3, 67.9, 106.4 | 143.5, 67.6, 106.3 | 143.6 67.6 106.8 |
| α, β, γ (°) | 90 94.5 90 | 90, 95.4, 90 | 90 95.4 90 |
| Resolution (Å) | 44.4 - 2.65 (2.74 - 2.65)* | 40.72-2.55 (2.64-2.55) | 40.93 - 3.15 (3.26 - 3.15) |
| *R*_merge_ | 0.125 (1.919) | 0.061 (1.349) | 0.119 (1.118) |
| *I* / σ*I* | 7.1 (0.69) | 11.91 (0.87) | 7.92 (0.84) |
| CC1/2 | 0.992 (0.407) | 0.999 (0.385) | 0.994 (0.425) |
| Completeness (%) | 98.9 (98.23) | 99.41 (99.40) | 98.08 (99.04) |
| Redundancy | 3.3 (3.4) | 3.3 (3.4) | 3.0 (3.0) |
|  |  |  |  |
| **Refinement** |  |  |  |
| Resolution (Å) | 44.4 - 2.65 (2.74 - 2.65) | 40.72-2.55 (2.64-2.55) | 40.93 - 3.15 (3.26 - 3.15) |
| No. unique reflections | 29,722 (2941) | 33,183 (3303) | 17,573 (1761) |
| *R*_work_ / *R*_free_ | 22.7/26.8 | 20.9/25.3 | 22.9/27.4 |
| No. atoms |  |  |  |
| Protein | 6,752 | 6,612 | 6,506 |
| Ligand/ion | 40 | 45 | 28 |
| Water | 0 | 9 | 0 |
| *B*-factors |  |  |  |
| Protein | 100.4 | 88.9 | 108.2 |
| Ligand/ion | 88.3 | 78.7 | 117.2 |
| Water |  | 65.5 |  |
| Ramachandran favored | 94.47 | 95.21 | 96.51 |
| Ramachandran outliers | 0.61 | 0.0 | 0.13 |
| Rotamer outliers | 0.53 | 0.41 | 0.0 |
| R.m.s. deviations |  |  |  |
| Bond lengths (Å) | 0.003 | 0.003 | 0.004 |
| Bond angles (°) | 0.53 | 0.59 | 0.56 |

*Values in parentheses are for highest-resolution shell.

Number of crystals used for each of the above structures=1
